# Connexin 30 controls astroglial polarization during postnatal brain development

**DOI:** 10.1242/dev.155275

**Published:** 2018-02-15

**Authors:** Grégory Ghézali, Charles-Félix Calvo, Laure-Elise Pillet, Flora Llense, Pascal Ezan, Ulrike Pannasch, Alexis-Pierre Bemelmans, Sandrine Etienne Manneville, Nathalie Rouach

**Affiliations:** 1 Center for Interdisciplinary Research in Biology, Collège de France, CNRS UMR 7241, INSERM U1050, Labex Memolife, PSL Research University, Paris 75005, France; 2 Doctoral School N°158, Pierre and Marie Curie University, Paris 75005, France; 3 Doctoral School N°562, Paris Descartes University, Paris 75006, France; 4 Institut Pasteur, CNRS UMR 3691, Cell Polarity, Migration and Cancer Unit, 25 Rue du Dr Roux, 75724 Paris Cedex 15, France; 5 Commissariat à l'Energie Atomique et aux Energies Alternatives (CEA), Département de la Recherche Fondamentale, Institut de biologie François Jacob, MIRCen, and CNRS UMR 9199, Université Paris-Sud, Neurodegenerative Diseases Laboratory, Fontenay-aux-Roses 92260, France

**Keywords:** Astrocytes, Connexin, Polarity, Development, Hippocampus, Mouse

## Abstract

Astrocytes undergo intense morphological maturation during development, changing from individual sparsely branched cells to polarized and tremendously ramified cells. Connexin 30, an astroglial gap-junction channel-forming protein expressed postnatally, regulates *in situ* the extension and ramification of astroglial processes. However, the involvement of connexin 30 in astroglial polarization, which is known to control cell morphology, remains unexplored. We found that connexin 30, independently of gap-junction-mediated intercellular biochemical coupling, alters the orientation of astrocyte protrusion, centrosome and Golgi apparatus during polarized migration in an *in vitro* wound-healing assay. Connexin 30 sets the orientation of astroglial motile protrusions via modulation of the laminin/β1 integrin/Cdc42 polarity pathway. Connexin 30 indeed reduces laminin levels, inhibits the redistribution of the β1-integrin extracellular matrix receptors, and inhibits the recruitment and activation of the small Rho GTPase Cdc42 at the leading edge of migrating astrocytes. *In vivo*, connexin 30, the expression of which is developmentally regulated, also contributes to the establishment of hippocampal astrocyte polarity during postnatal maturation. This study thus reveals that connexin 30 controls astroglial polarity during development.

## Abstract (precis)

**Summary:** Connexin 30 sets the orientation of astroglial motile protrusions during polarized migration *in vitro* and contributes *in vivo* to the establishment of hippocampal astrocyte polarity during postnatal maturation.

## INTRODUCTION

During early postnatal stages, individual sparsely branched astrocytes mature into extremely ramified cells to form interconnected cellular networks. The establishment of astrocytic networks relies especially on the timely tuned expression of gap-junction (GJ) subunit proteins, the so-called connexins (Cxs). Remarkably, the two main astroglial Cxs, Cx43 and Cx30, show different developmental expression patterns: in mice, expression of Cx43 starts around embryonic day 12, whereas that of Cx30 is detectable from postnatal day 16 (P16) (Kunzelmann et al., 1999). Strikingly, along with their well-known role in gap-junction (GJ)-mediated intercellular coupling, the functions of Cxs also include the formation of hemichannels (HCs) at the single cell level, which enables bidirectional exchanges with the extracellular space, as well as non-channel functions, which are mediated by Cx protein interactions or intracellular signaling. The isoform-specific sequence of the Cx intracellular C-terminal tail indeed contains multiple protein-binding motifs and thus serves as a multi-modal signaling platform for Cx-interacting partners (Herve et al., 2007). Consistently, proteomic analysis revealed that Cx43 functionally interacts with various cytoskeletal proteins, including actin and tubulin, which are involved *in vitro* in the formation of astroglial protrusions (Olk et al., 2010). Interestingly, Cx43 locally interacts with actin at the tip of astrocyte processes during the early stage of adhesion, as shown by combining atomic force microscopy with immunolabeling (Yamane et al., 1999). In addition, the non-channel functions of Cx43 and Cx26 are essential *in vivo* during embryonic neurodevelopment, notably by mediating intercellular adhesion between migrating cortical neurons and radial glia fibers (Elias et al., 2007). Such adhesion may rely on cross-junctional interactions between Cx-containing GJs and adhesion complexes (Xu et al., 2001, 2006).

In contrast and despite the late postnatal onset of Cx30 expression, little is known about the contribution of Cx30 to astroglial maturation during brain development. Similar to other Cxs, Cx30 interacts with the actin and tubulin cytoskeletons (He et al., 2009; Qu et al., 2009). In addition, our recent work shows that Cx30, via an unconventional function involving its C-terminal domain, controls postnatally in the hippocampus the extension and ramification of astroglial processes independently of GJ biochemical coupling and hemichannel activity (Pannasch et al., 2014). Remarkably, such regulation occurs at the whole-cell level as well as at perisynaptic sites, where it regulates the insertion of astroglial protrusions into synaptic clefts, and thereby directly sets synaptic glutamate levels through clearance. As local extension and ramification of fine processes relies on motility and polarity (Parsons et al., 2010), we here explored a role for Cx30 in the polarization of migrating astrocytes. We found that Cx30 expressed early on in astrocytes alters the reorientation of their protrusions, the microtubule organizing center (MTOC) and Golgi apparatus during polarized migration in the *in vitro* scratch assay. This effect is independent of GJ-mediated biochemical coupling and is mediated by modulation of a laminin/β1-integrin/Cdc42 signaling pathway (Etienne-Manneville and Hall, 2001; Peng et al., 2008). Cx30 indeed perturbed laminin levels and distribution in migrating astrocytes, the redistribution of β1-integrin receptors at leading edges of astroglial protrusions, and the polarized recruitment and activation of the polarity protein Cdc42 (Etienne-Manneville, 2008). Importantly, we also found that Cx30, the expression of which is developmentally regulated, contributed *in vivo* to the establishment of astrocyte polarity in the hippocampus during postnatal maturation, by setting the orientation of *stratum radiatum* (*s.r.*) astroglial processes. This study thus identifies Cx30 as an important regulator of astrocyte polarization during development.

## RESULTS

### Cx30 regulates the polarization of migrating astrocytes

**Fig. 1.**
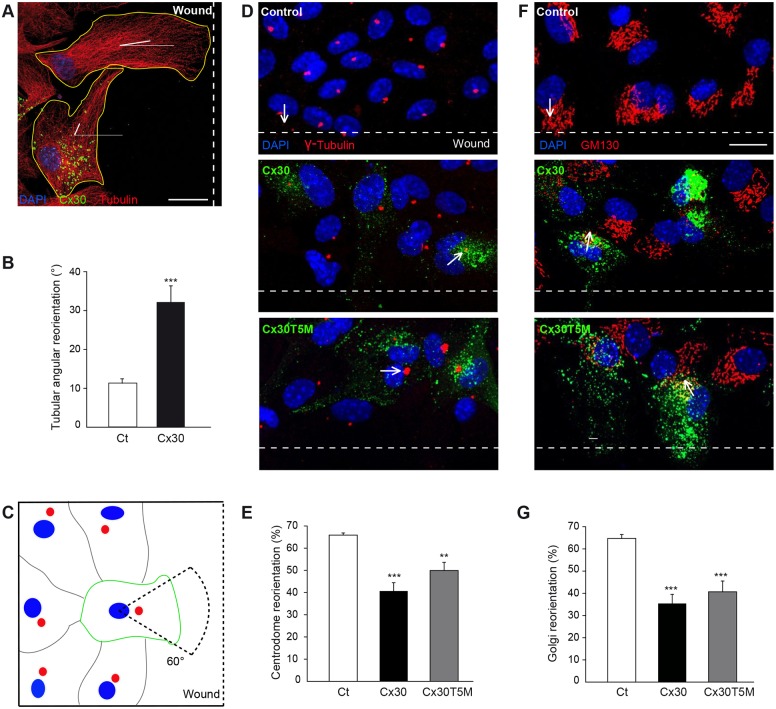
**Cx30 regulates the orientation of migrating astrocytes.** Primary astrocyte cultures were transfected with the Cx30-Venus plasmid. (A) 8 h after wounding, control and Cx30-expressing cells were immunolabeled for tubulin and the orientation of their tubulin fibers were measured. Scale bar: 20 µm. (B) The orientation of tubulin fibers was quantified in transfected and non-transfected cells as the mean angular variation to the perpendicular in relation to the wound [control (Ct), *n*=48 cells; Cx30, *n*=30 cells]. Cx30-expressing cells showed a greater angular deviation than control cells, suggesting that their cytoskeletal orientation was impaired. (C) Schematic depicting centrosome (red) reorientation of wound edge astrocytes (green) in front of the nucleus (blue) in the control condition. (D) 8 h after wounding, cells were immunolabeled for γ-tubulin (red) to mark the centrosome. The orientation of the nucleus-centrosome axis was measured. Arrows indicate the direction of the nucleus-centrosome axis. (E) Percentage of cells with their centrosome oriented perpendicularly (±45°) to the wound (Ct, *n*=313 cells; Cx30, *n*=187 cells; Cx30T5M, *n*=418 cells). Cx30 and Cx30T5M decreased the proportion of astrocytes with their centrosome properly reoriented. (F) 8 h after wounding, cells were immunolabeled for GM130 and the orientation of the nucleus-Golgi axis was measured. Scale bar: 20 µm. Arrows indicate the direction of the nucleus-Golgi axis. (G) Percentage of cells with their Golgi apparatus oriented perpendicularly (±60°) to the wound (Ct, *n*=352 cells; Cx30, *n*=153 cells; Cx30T5M, *n*=503 cells). Cx30 and Cx30T5M decreased the proportion of astrocytes with their Golgi properly orientated. Asterisks indicate statistical significance (***P<0.001; **P<0.01).

To investigate a role for Cx30 in the polarization of migrating astrocytes, we first used the scratch-induced migration assay in cultured astrocytes (Etienne-Manneville, 2006) transfected with a construct encoding Cx30 fused to the Venus YFP mutant protein (Cx30-Venus) (Beltramello et al., 2003). Cx30-Venus displayed proper cellular distribution and channel function, as it was targeted to cell membranes, formed gap junctional plaques and functional GJ channels, as shown by immunocytochemistry and the scrape loading tracer transfer technique (Fig. S1). Following scratching of the cell monolayer, wound-edge control astrocytes polarized and initiated directed migration by extending tubulin-rich protrusions perpendicularly to the wound (±11.2°±1.2, *n*=48 cells, Fig. 1A,B). Strikingly, expressing Cx30 impaired the proper orientation of migrating astroglial protrusions (with a mean variation of 32.2°±4.2, *n*=30 cells, Fig. 1A,B). This astrocyte polarization is also associated with the relocalization of the centrosome and the Golgi apparatus in front of the nucleus in the direction of the developing protrusion (Etienne-Manneville and Hall, 2001), as assessed by immunostaining for γ-tubulin and GM130, respectively (Fig. 1C). We found that Cx30 expression also altered the scratch-induced reorientation of the centrosome [% of polarized; Ct (control astrocytes): 65.9±1%, *n*=313 cells; Cx30 (Cx30 expressing astrocytes): 40.7±4%, *n*=187 cells, Fig. 1D,E] and Golgi apparatus (Ct: 64.7±2%, *n*=352 cells; Cx30: 35.4±4%, *n*=153 cells, Fig. 1F,G). Cx30-mediated regulation of astrocyte polarization did not involve GJ-mediated intercellular biochemical coupling, as the defect in cell polarity still occurred in astrocytes expressing the Cx30T5M construct (% of polarized cells assessed by reorientation of the centrosome: 49.9±3.7%, *n*=418 cells; Golgi apparatus: 40.7±4.8%, *n*=503 cells, Fig. 1D-G), a mutated form of Cx30 in which the replacement of a threonine by a methionine at position 5 of Cx30 inhibits intercellular biochemical coupling mediated by Cx30 GJ channels (Grifa et al., 1999; Schütz et al., 2010) (Fig. S1). These data demonstrate that Cx30 alters the polarization of migrating astrocytes independently of GJ-mediated intercellular biochemical coupling.

### Cx30 regulates the expression and distribution of laminin and sets the polarized distribution of β1 integrin in migrating astrocytes

**Fig. 2.**
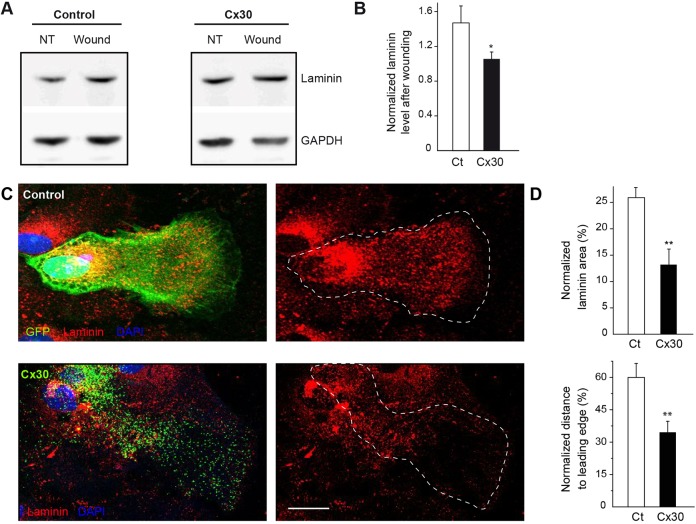
**Cx30 regulates the secretion of laminin in migrating astrocytes.** (A) Immunoblot detection of laminin expression in control (Ct) or Cx30-expressing (Cx30) astrocytes before (no treatment, NT) or after wounding (wound, 6 h). GAPDH was used as a loading control. (B) Quantitative analysis of laminin expression (*n*=6 cultures). Laminin expression values after wounding were normalized to levels before wounding (NT) in Ct and Cx30-expressing astrocytes. Cx30 prevented the increase in relative laminin levels after wounding. (C) Primary astrocytes were transfected with GFP (control) or Cx30-venus (Cx30). 24 h after wounding, cells were immunolabeled for laminin. Scale bar: 20 μm. (D) Quantitative analysis of laminin expression (area occupied by laminin puncta normalized to total cell area) and axial distribution (distance of laminin puncta to leading edge normalized to cell length). Cx30 impaired the proper recruitment of laminin toward the leading edge (Ct, *n*=14 cells; Cx30, *n*=15 cells). Asterisks indicate statistical significance (*P<0.05, **P<0.01).

Migrating astrocytes contribute to the deposition of extracellular matrix (ECM) molecules, including laminins, which are essential for integrin-mediated cell polarization (Peng et al., 2008). We thus investigated whether astroglial Cx30 regulates the expression of laminin upon wounding. Immunoblotting confirmed that laminin expression was upregulated in control migrating astrocytes after wounding (Fig. 2A,B), as previously reported (Peng et al., 2008). We found that Cx30 expression inhibited the wound-induced upregulation of laminin expression in cultured astrocytes (normalized laminin expression value in astrocytes after wounding compared with before wounding; Ct: 1.47±0.20, *n*=6; Cx30: 1.06±0.08, *n*=6 cultures, Fig. 2A,B). Consistent with these data, immunocytochemistry revealed that Cx30 reduced total laminin levels in wounded astrocytes (Fig. 2C,D) through a decrease in both the number (Ct: 0.125±0.08 per µm^2^, *n*=14 cells; Cx30: 0.074±0.011 per µm^2^, *n*=15 cells, Mann–Whitney, *P*<0.01) and size of laminin puncta (Ct: 2.068±0.078 µm^2^, *n*=14 cells; Cx30: 1.603±0.197 µm^2^, *n*=15 cells, Mann–Whitney, *P*<0.05). Remarkably, during polarized migration, laminin puncta extended up to the leading edge of astroglial protrusions where they accumulated, as shown by immunocytochemistry in GFP-labeled cells (Fig. 2C). We found that Cx30 not only decreased total laminin levels, but also inhibited the polarized distribution of laminin puncta toward the leading edge of migrating astrocytes (Fig. 2C,D).

**Fig. 3.**
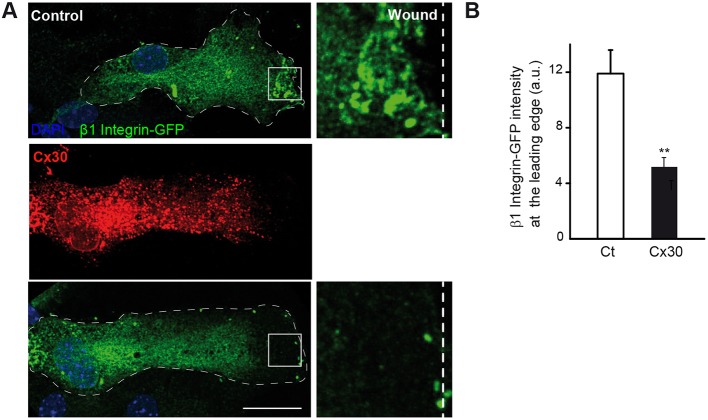
**Cx30 controls the polarized distribution of β1 integrin receptors in migrating astrocytes.** (A) Primary astrocytes were transfected with β1 integrin-GFP and Cx30. 8 h after wounding, cells were immunolabeled for Cx30 and the intensity of the β1 integrin-GFP signal at the leading edge was measured. Scale bar: 20 µm. (B) Quantitative analysis of the β1 integrin-GFP signal intensity at the leading edge. Cx30 sharply reduced the recruitment of β1 integrin-GFP at the leading edge (Ct, *n*=15 cells; Cx30, *n*=9 cells). Asterisks indicate statistical significance (**P<0.01).

Laminin locally binds receptors, including β1 integrins, which are trafficked to the cell leading edge where they are essential for proper astrocyte polarization (Peng et al., 2013). To explore whether Cx30 regulates the translocation of β1 integrin to the leading edge, we transfected astrocytes with both GFP-tagged β1 integrin and Cx30. Cx30 prevented the polarized recruitment of β1 integrin-GFP at the cell wound edge [β1 integrin-GFP intensity at the protrusion edge (a.u.): Ct: 11.9±1.7, *n*=15 cells; Cx30: 5.2±0.7, *n*=9 cells, Fig. 3A,B]. Moreover, Cx30 occasionally induced accumulation of β1 integrin-GFP in the perinuclear area. Altogether, these data demonstrate that Cx30 controls the level of laminin expression and distribution, as well as the subcellular trafficking of β1 integrin during astrocyte polarization.

### Cx30 controls the activation and polarized recruitment of Cdc42

**Fig. 4.**
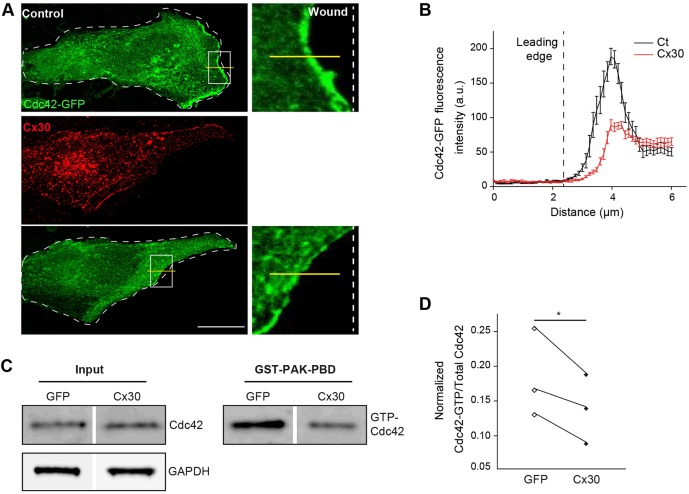
**Cx30 controls the recruitment and activation of Cdc42 in migrating astrocytes.** (A) Primary astrocyte cultures were transfected with Cx30 and GFP-Cdc42. 8 h after wounding, cells were immunostained for Cx30 and the intensity of the GFP-Cdc42 signal at the leading edge was measured. Scale bar: 20 µm. (B) Quantitative analysis of the linear intensity profile of GFP-Cdc42 at the leading edge. Cx30 reduced significantly the recruitment of GFP-Cdc42 at the leading edge (Ct, *n*=19 cells; Cx30, *n*=15 cells). (C) Cdc42 pull-down activation assay in astrocytes transfected with Cx30 or GFP 30 min after wounding. Immunoblots showing Cdc42 protein levels in the total (Input) and GTP-bound fractions (GST-PAK-PBD). GAPDH (total) and Ponceau staining (GTP) were used as loading controls. (D) Quantitative analysis of Cdc42 activation (*n*=3 cultures). Cdc42-GTP values were normalized to Cdc42 levels in the total fractions. Cx30 inhibited Cdc42 activation. Asterisk indicates statistical significance (*P<0.05, paired *t*-test).

Integrin-mediated signaling promotes astrocyte polarization through the rapid activation and polarized recruitment of the small Rho-GTPase Cdc42 at the leading edge of migrating astrocytes (Etienne-Manneville and Hall, 2001). We thus assessed whether Cx30 modulates the recruitment and activation of Cdc42 in astrocytes expressing a green fluorescent protein (GFP)-tagged Cdc42 together with the Cx30 protein (Etienne-Manneville and Hall, 2001). We found that GFP-Cdc42 accumulated at the leading edge of control migrating astrocytes (Fig. 4A,B), as previously reported (Osmani et al., 2006). In contrast, Cx30 expression prevented the accumulation of Cdc42 at cell front (Ct: 188.5±11.9 a.u., *n*=19 cells; Cx30: 89.3±7.6 a.u., *n*=15 cells; Fig. 4A,B). The activation of the Rho-GTPase Cdc42 is mediated by the switch from a GDP-bound to a GTP-bound state (Sinha and Yang, 2008). We found that Cx30 also inhibited the wound-induced activation of Cdc42 by ∼25%, as assessed by a pull-down activation assay (GTP-Cdc42 as % of the total Cdc42 detected: GFP: 18.3±3.7%; Cx30: 13.8±2.9, *n*=3 cultures, Fig. 4C,D). These data suggest that Cx30 influences astrocyte polarization by altering the recruitment and activation of Cdc42 at cell wound edge.

### Synaptic levels of laminin are set by astroglial Cx30

**Fig. 5.**
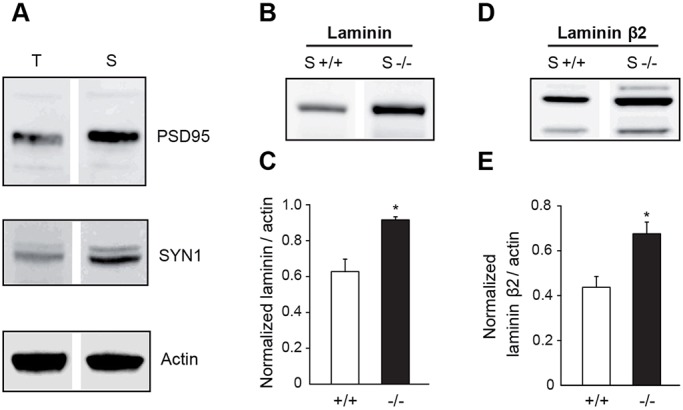
**Cx30 regulates hippocampal synaptic levels of laminin proteins.** (A) Western blot detection of pre- (synapsin I, SYN1) and post- (PSD95) synaptic proteins showed an enrichment of plasma membrane synaptic proteins in crude synaptosomal membrane fraction (S) compared with total hippocampal fraction (T), whereas actin protein levels were clearly similar in both fractions. (B) Immunoblot detection of laminin expression in synaptosomal membrane fractions from mouse hippocampi. (C) Histogram showing the quantitative analysis of normalized laminin expression levels (+/+, *n*=3 mice; −/−, *n*=3 mice). The loss of Cx30 expression increased significantly the synaptic levels of laminin. (D) Immunoblot detection of laminin β2 protein levels in crude hippocampal membrane fractions. (E) Quantitative analysis of laminin β2 normalized expressions. Hippocampal synapses lacking Cx30 upregulated laminin β2 expression. In both experiments, actin was used as a loading control. Asterisks indicate statistical significance (*P<0.05). S+/+, wild-type mice; S−/−, *Cx30^−/−^* mice.

During early postnatal development, protoplasmic astrocytes extend countless fine protruding processes that migrate to virtually fill the entire neuropil and thus reach the vicinity of synapses. The ECM component laminin β2, which is deposited on the surface of synapses and is essential for building-up and maintaining the proper synapse architecture (Egles et al., 2007), has been reported to be locally released by astrocyte processes (Hunter et al., 1992). As we found Cx30 to negatively regulate the deposition of laminin proteins in cultured astrocytes, we explored the possibility that Cx30 may also control *in vivo* the expression of laminins at synaptic sites. To do so, we prepared synaptosomal extracts from hippocampi of wild-type juvenile mice (+/+, Fig. 5A) or mice lacking the Cx30 protein (*Cx30^−/−^, −/−*), and further performed laminin proteins immunoblot assays. We observed that control hippocampal synaptosomal extracts contained laminins, especially laminin β2 (Fig. 5B,D) as previously demonstrated (Egles et al., 2007). Interestingly, we observed that the absence of Cx30 induced a net increase in the synaptic level of laminins [normalized protein levels (a.u.): +/+: 0.63±0.07; −/−: 0.92±0.02, *n*=3 mice, Fig. 5B,C], notably laminin β2 [normalized protein levels (a.u.): +/+: 0.44±0.05; −/−: 0.68±0.05, *n*=3 mice, Fig. 5D,E]. In all, these data demonstrate that Cx30 mediates hippocampal synaptic laminin downregulation *in vivo*.

### Cx30 contributes *in vivo* to the morphological and functional polarity of astrocytes during postnatal development

**Fig. 6.**
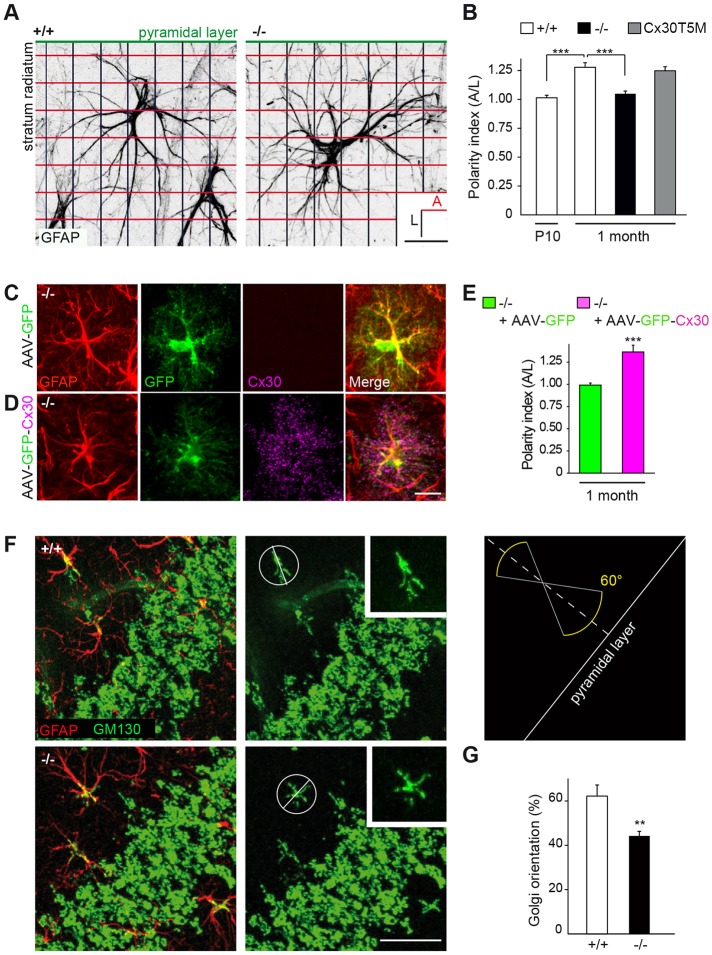
**Cx30 sets the polarity of hippocampal astrocytes *in vivo*.** (A) Schematic representation of grid-baseline analysis for orientation quantification of GFAP-labeled CA1 *s.r.* astrocytes. Scale bar: 20 µm. (B) Quantitative analysis of astrocytes polarity with respect to the pyramidal cell layer. Cx30 was found to be required for the proper perpendicular orientation of developing astrocytes (+/+ P10, *n*=43 cells; +/+ 1 month, *n*=44 cells; −/− 1 month, *n*=49 cells; Cx30T5M 1 month, *n*=47 cells). (C,D) Cx30 expression in astrocytes from the hippocampal CA1 *s.r.* region of *Cx30^−/−^* mice (1-month-old) infected with AAV-GFP (C) or AAV-GFP-Cx30 (D), as shown by GFAP, GFP and Cx30 labeling in hippocampal sections. Scale bar: 15 µm. (E) Quantitative analysis of astrocytes polarity in −/− mice showing that wild-type polarity is rescued in *s.r.* astrocytes by restoring Cx30 expression (*n*=32 cells), but not by expressing GFP alone (*n*=34 cells). (F) Immunohistochemical labeling of GM130 (green) and GFAP (red) in the CA1 area of the hippocampus. The diagram illustrates the quantification used to estimate the angular orientation of astrocyte Golgi with regards to the pyramidal layer. Scale bar: 30 µm. (G) Percentage of cells with their Golgi apparatus oriented perpendicularly (±30°) to the pyramidal layer (+/+, *n*=216 cells; −/−, *n*=262 cells). Cx30 deficiency decreased the proportion of CA1 astrocytes with their Golgi oriented perpendicularly to the pyramidal layer. Asterisks indicate statistical significance (***P<0.001; **P<0.01).

**Fig. 7.**
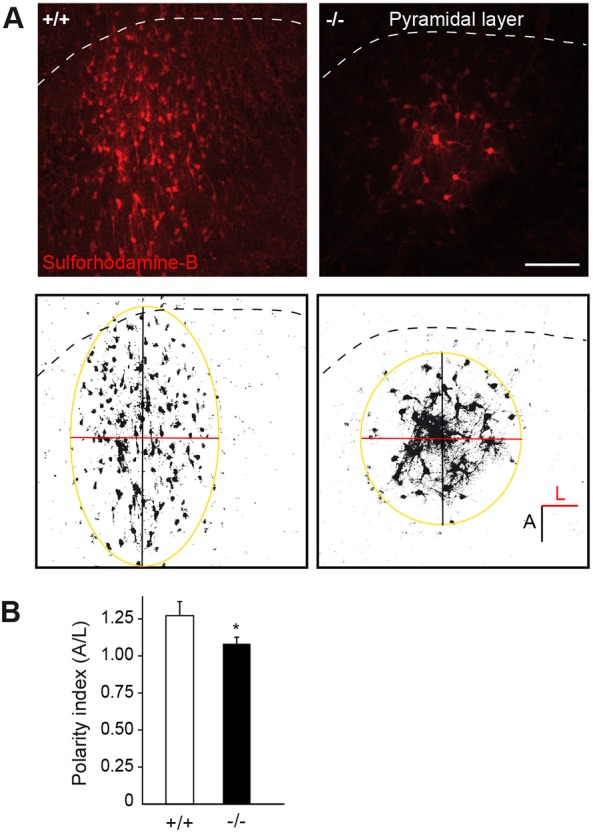
**Cx30 sets the spatial properties of intercellular communication in astroglial networks.** (A) Representative images of GJ-mediated coupling between CA1 astrocytes visualized by diffusion of sulforhodamine B via patch clamp whole-cell recording. Schematic showing the method used for estimating the spatial properties of CA1 astroglial networks. Scale bar: 100 µm. (B) Graph summarizing the spatial properties of CA1 astroglial networks in the form of polarity indexes. Cx30 is required for the anisotropic coupling of CA1 *s.r.* astrocytes (+/+, *n*=8 slices; −/−, *n*=10 slices). Polarity index ratio larger than one indicates preferential perpendicular orientation towards the pyramidal layer. Asterisk indicates statistical significance (*P<0.05).

Starting at the third week of postnatal development, i.e. at the time of hippocampal synaptogenesis, CA1 astrocytes of the *s.r.* change the orientation of their glial fibrillary acidic protein (GFAP)-rich stem processes to polarize perpendicularly to the pyramidal cell layer (Nixdorf-Bergweiler et al., 1994). As we show here *in vitro* that Cx30 regulates the orientation of astrocyte motile protrusions (Fig. 1A), we took advantage of the developing hippocampus to investigate whether Cx30 controls the *in vivo* reorientation of astroglial processes occurring during postnatal maturation. To do so, we performed GFAP immunohistochemistry in the hippocampus of Cx30 knockout mice (*Cx30^−/−^*) at two developmental stages [postnatal day 10 (P10) and 30 (1 month)]. Moreover, to estimate the preferential orientation of *s.r.* astrocytes with regards to the pyramidal layer, we measured a polarity index corresponding to the ratio of crossing points between GFAP-positive processes and parallel or perpendicular axis to the pyramidal layer (Fig. 6A). We found in wild-type mice that P10 astrocytes did not show any preferential orientation (polarity index: 1.01±0.02, *n*=44 cells, Fig. 6A,B), whereas astroglial cells from 1-month-old mice preferentially displayed an axial orientation (polarity index: 1.28±0.04, *n*=43 cells, Fig. 6A,B), thereby confirming previous observations (Nixdorf-Bergweiler et al., 1994). Strikingly, we observed in 1-month-old *Cx30^−/−^* mice that astrocytes lost their preferential orientation regarding the pyramidal layer (polarity index: 1.05±0.02, *n*=49 cells). This modulation of astroglial cell polarity was independent of GJ-mediated intercellular biochemical coupling, as astroglial polarity was intact in astrocytes from 1-month-old Cx30T5M mutant mice with defective Cx30 channels (Fig. 6B). Remarkably, we found that restoring *in vivo* Cx30 expression selectively in astrocytes from the hippocampus of *Cx30^−/−^* mice at P15 using adenoviral vectors (Fig. 6C,D and Fig. S3) was sufficient to fully rescue a normal polarity phenotype in *s.r.* astrocytes from 1-month-old animals, whereas GFP infection alone had no effect (polarity index: −/−+GFP: 0.99±0.02, *n*=34 cells; −/−+Cx30: 1.36±0.07, *n*=32 cells, Fig. 6E). These data indicate that postnatal Cx30 expression contributes to the establishment of astroglial polarity in the developing hippocampus. We also performed analysis of CA1 *s.r.* astrocyte orientation by immunohistochemistry of the Golgi marker GM130 in 1-month-old wild-type and *Cx30^−/−^* mice. We found that the Golgi apparatus of astrocytes formed bundles of stretched and polarized vesicular compartments, having eventual small branches that filled the base of GFAP^+^ astrocyte processes (Fig. 6F). Similarly, we observed that disruption of Cx30 reduced the percentage of cells that properly orientated their Golgi perpendicularly to the pyramidal layer (+/+: 62.2±5.0, *n*=262 cells; −/−: 44.2±2.0%, *n*=266 cells, Fig. 6F,G). These findings show that Cx30 is a crucial factor that regulates the orientation of hippocampal astrocytes *in vivo* during postnatal development. Interestingly, it has recently been postulated that the radial orientation of CA1 astrocytes may control the spatial properties of GJ-mediated intercellular coupling (Anders et al., 2014). As Cx30 regulates the orientation of CA1 *s.r.* astrocytes established during development in the hippocampus, we investigated whether this translated at the functional level into the anisotropy of intercellular communication in astroglial networks. To do so, we dialyzed a GJ channel-permeable dye (sulforhodamine-B) in CA1 *s.r.* astrocytes from hippocampal slices via patch-clamp whole-cell recording. We found that wild-type CA1 astroglial GJ-mediated networks were preferentially oriented perpendicularly to *stratum pyramidale* (*s.p.*) (with a polarity index of 1.27±0.10, *n*=8 slices, Fig. 7A,B). In contrast, Cx30-deficient astrocyte networks extended with no preferential orientation (polarity index: 1.08±0.04, *n*=10 slices, Fig. 7A,B). This indicates that Cx30 functionally regulates the spatial properties of intercellular communication in astroglial networks. Altogether, these data show that Cx30 controls *in vivo* the establishment of astrocyte polarity during postnatal development.

## DISCUSSION

The present study shows that Cx30 modulates a laminin/β1 integrin-mediated signaling cascade that controls the reorientation of astroglial migrating protrusions, especially contributing to the postnatal morpho-functional maturation of hippocampal astrocytes.

Here, we have found *in vitro* that Cx30 expression sets the polarization of astroglial protrusions independently of GJ-mediated biochemical coupling. This is reminiscent of an unconventional function of Cx30 that we recently identified in the hippocampus, and which regulates excitatory synaptic transmission by controlling the ramification and insertion of astroglial processes into synaptic clefts (Pannasch et al., 2014). Indeed, by showing that perisynaptic astroglial processes lacking Cx30 literally penetrate synaptic clefts, we revealed that Cx30, via its C-terminal domain and independently of its hemichannel activity and GJ biochemical coupling, is a molecular determinant of astroglial synapse coverage controlling synaptic efficacy and hippocampal-based memory (Pannasch et al., 2014). As Cx30 interacts with elements of the cytoskeleton, such as actin and tubulin (He et al., 2009; Qu et al., 2009), it most likely regulates the dynamic and local changes in astroglial polarity and morphology through its participation in intracellular molecular complexes. Here, we have found *in vitro* that Cx30 inhibits the upregulation of laminin expression, as well as its polarized distribution toward the leading edge of migrating astrocytes. Remarkably, knocking down Cx43 in human fibroblasts also alters expression of ECM molecules, including laminins, as shown by transcriptional and immunoblot analysis (Esseltine et al., 2015). Interestingly, local regulation of laminin expression has been shown to be the priming event of the β1 integrin-mediated polarity pathway (Frank and Carter, 2004), and we also here reveal that Cx30 regulates the polarized targeting of β1 integrin to the cell wound edge. In addition, our data show that Cx30 controls the recruitment and activation of Cdc42 at the cell leading edge. This finding is consistent with previous studies, showing that Cxs, in particular Cx43, control the activation of small Rho GTPases, such as RhoA, Rac1 and Rap1 (Machtaler et al., 2011; Mendoza-Naranjo et al., 2012). The control of Cdc42 activity by Cx30 may dictate the reorientation of the astrocyte centrosome and Golgi. Noteworthy, Cx30 inhibits the rapid activation of Cdc42 shortly after wounding. Therefore, the lack of laminin deposition observed in Cx30-overexpressing astrocytes may actually result from the early downregulation of Rho GTPase activity, as previously reported (Wu et al., 2006).

Cx30 starts being expressed around postnatal day 16 in mouse hippocampus, which is exactly the time of synaptogenesis and synaptic pruning (Kunzelmann et al., 1999). The remodeling of the neuropil ECM meshwork, especially laminins, has been associated with long-term synaptic plasticity (Calabresi et al., 2000; Nakagami et al., 2000) and activity-dependent remodeling of pyramidal cell dendrites (Mataga et al., 2004). Here, we report that Cx30 downregulates the deposition of laminins in hippocampal synaptosomal extracts, and particularly the synapse-enriched isoform laminin β2. Our data thus suggest that Cx30 regulates the ECM-mediated plasticity of neuroglial synaptic circuits in the developing hippocampus. This hypothesis is consistent with the fact that *Cx30^−/−^* mice display impaired hippocampal long-term synaptic plasticity and hippocampus-based contextual fear memory (Pannasch et al., 2014). Noteworthy, we demonstrate that Cx30, independently of GJ-mediated biochemical coupling, is also necessary during hippocampal development for the reorientation of CA1 *s.r.* astrocyte processes along pyramidal cell apical dendrites: the locus of excitatory synapses. Cx30 modulation of laminin signaling likely sets the reorientation of migrating protrusions by regulating astroglial polarization. Noteworthy, we identify that Cx30-mediated regulation of astroglial polarity is multifaceted, as Cx30 overexpression *in vitro* inhibited the polarity in migrating astrocytes in response to a scratch, whereas Cx30 disruption *in vivo* abolished the establishment of hippocampal astrocyte polarity during postnatal development. One can hypothesize that astrocyte polarity may be tightly regulated by Cx30 protein levels, implying an expression level-dependent mechanism. It is indeed possible that a defined and ‘optimal’ Cx30 expression level is necessary for proper astroglial polarity, as is the case for Cdc42 or αPKC, which perturb polarity when inhibited as well as when overactivated (Etienne-Manneville and Hall, 2001). Such a general feature applies for several other cellular processes, such as membrane extension and migration, which are tightly regulated by defined level of adhesion molecules (DeMali and Burridge, 2003). Cx30 may also be required for the pruning of astroglial processes, which sets proper cell polarity. Astrocytes lacking Cx30 have indeed been shown to display larger GFAP domain area, elongated processes and enhanced ramification (Pannasch et al., 2014), all of which may be due to a reduced developmental pruning. Interestingly, we found that the developmental reorientation of CA1 astrocyte processes is accompanied by a similar Cx30-dependent alignment of the Golgi apparatus along the axis of pyramidal cell dendrites. Therefore, it may be hypothesized that this subcellular reorganization supports the acquisition of polarized secretory competence by mature astroglial cells. Interestingly, the loss of radial orientation in *s.r.* astrocytes from juvenile *Cx30^−/−^* mice has functional consequences, as it is associated with a defect in the spatial properties of astroglial networks. This suggests that Cx30, by allowing proper astrocyte orientation, shapes the spatial properties of functional GJ coupling in CA1 *s.r.* astrocytic networks. This observation corroborates a previous study proposing that the preferential anisotropic coupling of CA1 hippocampal astrocytes relies upon the polarized orientation of their processes (Anders et al., 2014). Remarkably, signaling molecules regulating cell polarity, such as laminin/β1integrin/Rho GTPases also modulate *in vitro* the permeability of GJ channels and thus the extent of astroglial networks (Derangeon et al., 2008; Rouach et al., 2006). Noteworthy, several physiological and pathological conditions alter the polarity of astrocytes in the *s.r.* of the hipppocampal CA1 region. In particular, opposite changes in astrocyte polarity occur with physical exercise (Saur et al., 2014) or enriched environment (Viola et al., 2009) and post-traumatic stress disorder (Saur et al., 2016), suggesting that polarity likely plays an important role in the function of astrocytes during physiological and pathological processes.

Altogether, these data reveal the role of Cx30 in the polarity of astrocytes through a laminin-mediated signaling pathway. Interestingly, wiring of synaptic networks during postnatal stages relies on the remodeling of the extracellular matrix, and concurs with the onset of Cx30 expression. Cx30 thus likely contributes in the hippocampus to the structural maturation of local astroglial circuits within neuronal functional units.

## MATERIALS AND METHODS

### Animals

Experiments were carried out according to the guidelines of the European Community Council Directives of January 1st 2013 (2010/63/EU) and of the local animal welfare committee (certificate A751901, Ministère de l'Agriculture et de l'Alimentation). All efforts were made to minimize the number of animals used and their suffering. Mice (*Mus musculus*) were group housed on a 12 h light/dark cycle. *Cx30^−/−^* (Teubner et al., 2003) and *Cx30^T5M/T5M^* mice (Schütz et al., 2010) were generated as previously described. All mice were backcrossed to the C57BL6 background. Juvenile mice of both sexes and littermates were used at postnatal days 21-30, unless otherwise stated.

### Antibodies, immunohistochemistry and immunoblotting

All the antibodies used in this study are commercially available and have been validated in previous studies, as reported by the suppliers. The following primary antibodies were used: Cx30 rabbit polyclonal (1:500, 71-2200, Zymed Laboratories), Cx43 mouse monoclonal (1:500, 610-062, BD Biosciences), GFAP rabbit polyclonal (1:2000, G3893, Sigma), GFP chicken polyclonal (1:500, AB13970, Abcam), α-tubulin rabbit polyclonal (1:10,000, T6199, Sigma), β-actin mouse monoclonal (1:10,000, A5316, Sigma), PSD95 mouse monoclonal (1:500, 610495, BD Biosciences), synapsin I mouse monoclonal (1:10,000, 106 011, Synaptic Systems), GM130 mouse monoclonal (1:500, 610822, BD Biosciences), γ-tubulin mouse monoclonal (1:500,T6557, Sigma), laminin rabbit polyclonal (1:1000, L9393, Sigma), laminin β2 mouse monoclonal (1:1000, sc-59980, Santa Cruz), Cdc42 mouse monoclonal (1:150, ACD03, Cytoskeleton), paxillin mouse monoclonal (1:200, 610569, BD Biosciences), vinculin (1:200, ab18058, Abcam), MAP2 mouse monoclonal (1:400, M4403, Sigma), IB4-647 (1:100, I32450, Invitrogen). The HRP-conjugated primary rabbit anti-GAPDH antibody (1:10,000, ab9385, Abcam) has been used as loading control. The following HRP-conjugated secondary antibodies were used: goat anti rabbit IgG (1:2500, sc-2004, Santa Cruz) and goat anti-mouse IgG (1:2500, sc-2005, Santa Cruz). The following fluorescent dye-conjugated secondary antibodies were used in appropriate combinations: goat anti-mouse IgG conjugated to Alexa 488 or 647 (1:2000, A-11001, A-21235, Thermo Fisher), goat anti-rabbit IgG conjugated to Alexa 488 or 555 (1:2000, A-11034 or A-21429, Thermo Fisher) and goat anti-chicken conjugated to Alexa 488 (1/2000; A-11039, Thermo Fisher).

Immunohistochemistry and quantifications were performed as previously described (Koulakoff et al., 2008). Briefly, cryostat brain slices were fixed for 10 min at room temperature with 4% paraformaldehyde (PFA) then washed three times with phosphate buffered saline (PBS) and pre-incubated 1 h with PBS-1% gelatin in the presence of 1% Triton-X100. Brain slices were then stained overnight at 4°C with primary antibodies and washed in PBS three times. Appropriate secondary antibodies were finally applied for 1-2 h at room temperature.

After several washes, brain slices were mounted in fluoromount and examined with an inverted confocal laser-scanning microscope (TCS SP5, Leica). Stacks of consecutive confocal images taken with a 63× objective at 600-1000 nm intervals were acquired sequentially with two lasers (argon 488 nm and helium/neon 543 nm) and *z* projections were reconstructed using ImageJ software. Alternatively, primary astrocyte cultures were fixed at room temperature with 4% paraformaldehyde for 10 min, washed three times with PBS and incubated 1 h with 10% non-immune goat serum (Zymed) in the presence of 0.1% Triton-X100 before proceeding as described previously.

Western blotting and quantification were performed as previously described (Pannasch et al., 2011). Shortly, cells were collected with a folded pipette tip (200 µl) in a small volume of PBS containing a cocktail of protease inhibitors (Boehringer), phosphatases inhibitors (β-glycerophosphate, 10 mM) and orthovanadate (1 mM), to which Laemmli 5× buffer was added. Samples were sonicated, boiled for 5 min and loaded on 10% or 4-12% polyacrylamide gels. Proteins were separated by electrophoresis and transferred onto nitrocellulose membranes. Membranes were saturated with 5% fat-free dried milk in triphosphate buffer solution and incubated overnight at 4°C with primary antibodies. They were then washed and exposed to peroxidase-conjugated secondary antibodies. Specific signals were revealed with the chemiluminescence detection kit (ECL, GE Healthcare). Semi-quantitative densitometric analysis was performed after scanning the bands with ImageJ software.

### Primary astrocyte cultures and scratch-induced migration assay

Primary cortical astrocyte cultures (Fig. S2A) were prepared as previously described (Koulakoff et al., 2008). Briefly, brains were removed from OF1 newborn pups (P1-P3) and the cortices were dissected in cold PBS-glucose (33 mM). Meninges were carefully withdrawn and cortices were mechanically dissociated with a flame-polished Pasteur pipette in PBS-glucose. Cells were seeded onto poly-ornithine-coated glass coverslips in DMEM containing 10% fetal calf serum, 10 U/ml penicillin and 10 µg/ml streptomycin (Gibco), and incubated at 37°C, 5% CO_2_. After 1 week, once cells have reached confluency, 1 µM of cytosine-arabinoside was added to the cell culture for 3 days to eliminate proliferating microglial cells. Medium was then changed every 3 days and cells were used after 2-3 weeks in culture. Astrocytes maintained for 2-3 weeks in culture express Cx43, but not Cx30 (Fig. S2B,C), as previously shown (Kunzelmann et al., 1999; Koulakoff et al., 2008).

Astrocytes were transfected 24 h before wounding with plasmids for either GFP or Cx30, Cx30-venus (Beltramello et al., 2003), Cdc42-GFP (Etienne-Manneville and Hall, 2001) or β1 integrin-GFP (human β1 integrin, provided by F. Coumailleau, Pasteur Institute, Paris, France). The medium was changed 12 h before performing scratch-induced migration assays. Confluent astrocytes were wounded by tidily scraping monolayers with a 200 µl pipette tip (≈300 µm in width). For biochemical analysis, several wounds were made in multiple directions. Cells were then fixed with 4% PFA and washed three times in PBS before proceeding with immunostaining as described above.

### Imaging analysis of astrocytes reorientation

*In vitro* tubulin fibers reorientation was assessed 8 h after wounding. Wounded astrocyte cultures were fixed with PFA and immunostained for tubulin. Confocal *z*-stack images of individual wound-edge astrocytes were analyzed using the ImageJ plug-in FibrilTool (Boudaoud et al., 2014). Briefly, regions of interest were drawn around a single-cell tubulin cytoskeleton and the average fibril orientation provided by the plug-in was compared with the actual angle of the axis perpendicular to the scratch (with regard to the horizontal plane).

*In vitro* centrosome and Golgi apparatus reorientation was determined as previously described (Etienne-Manneville, 2006). Shortly, 8 h after scratching, astrocytes were fixed with 4% PFA and immunolabeled for γ-tubulin (centrosome) or GM130 (Golgi), and DAPI was added to the secondary antibodies to label the nuclei. Wound-edge astrocytes with their centrosome or Golgi orientated perpendicularly (±45° and ±60°, respectively) to the scratch were considered properly polarized. Random orientation was thus scored at 25% for the centrosome and 33% for the Golgi.

*In vivo* GFAP-positive process reorientation was analyzed as previously described (Nixdorf-Bergweiler et al., 1994). Confocal *z*-stacks images of GFAP-labeled CA1 *s.r.* subfields were processed for grid-base line analysis using the ImageJ software. In short, the pyramidal cell layer was laid down on the top side of the image before setting up an overlay grid made of horizontal/vertical lines delimitating 100 µm^2^ squares. The number of intersections between GFAP-expressing astrocytic processes and grid lines was then measured. Intersections with horizontal lines reflected processes orientated perpendicularly to the pyramidal layer (thus displaying the so-called axial orientation), whereas processes crossing vertical lines were scored with a lateral orientation towards the pyramidal cell layer. A ratio between axial/lateral processes was finally measured, which reflects the polarity index of the cell. A polarity index of 1 indicates no polarity, whereas an index larger than 1 indicates preferential perpendicular orientation toward the pyramidal layer.

The protocol used for analysis of *in vivo* Golgi apparatus reorientation was directly adapted from the method of scoring Golgi repositioning upon scratching (Etienne-Manneville, 2006). Confocal *z*-stack images of GM130-labeled CA1 *s.r.* subfields were processed for angular orientation analysis using the orientation ImageJ plug-in. Succinctly, regions of interest were drawn around individual Golgi apparatus from CA1 astrocytes facing the pyramidal layer, and the orientation values provided were compared with those perpendicular to the pyramidal cell layer. Astrocytes that had their Golgi aligned with this axis (±30°, in both directions) to the scratch were considered properly polarized. Random orientation was thus scored at 33%.

The spatial properties of GJ-mediated inter-astrocytic coupling were analyzed using the ImageJ software. Confocal *z*-stack thresholded images of the sulforhodamine-B-labeled CA1 *s.r.* astrocyte networks were manually delimited with best-fit ellipses. The length of the major/minor ellipse axis were measured and its ratio (major/minor) provided an estimation of the polarity (or anisotropy) of astroglial networks. A polarity index value larger than one indicated preferential perpendicular orientation with respect to the pyramidal layer.

### Imaging analysis of protein relocalization

Analysis of Cdc42 relocalization was adapted from Osmani et al. (2010). In short, primary astrocytes were transfected with GFP-Cdc42 (together with Cx30), given that no Cdc42 antibody is currently available for immunolabeling. Eight hours after scratching, astrocytes were fixed with 4% PFA and immunostained for Cx30. The linear intensity profile of GFP-Cdc42 at the leading edge of migrating astrocytes was quantified using ImageJ software, and compared between control and Cx30-expressing cells.

Similarly, astrocyte monolayers were transfected with β1 integrin-GFP (provided by F. Coumailleau) and Cx30. 8 h after wounding, then cells were fixed with 4% PFA and immunolabeled for Cx30. Regions of interest were drawn at the leading edge of migrating astrocytes and the intensity of β1 integrin-GFP was quantified using ImageJ software and compared with control cells.

### Imaging analysis of laminin puncta

The analysis of laminin puncta number and size, as well as axial distribution was adapted from a previous study (Camand et al., 2012). Briefly, confocal images of laminin immunostained astrocyte cultures were binarized and thresholded using the ImageJ software. Regions of interest encompassing laminin puncta up to the leading edge were then drawn in front of the nucleus of individual cells such that the average number of laminin puncta could be measured using the Analyze Particle tools of ImageJ. The pixel size values (min/max) were especially set to include most laminin puncta. Similarly, the mean area occupied by laminin puncta was quantified after thresholding of laminin immunofluorescence confocal images using the ImageJ software. Finally, each laminin puncta centroid was projected on an axis running from the nucleus to the cell leading edge and the normalized mean projected distances were calculated using the ImageJ software.

### Cdc42 pull-down activation assay

The Cdc42 pull-down assay was adapted from a commercial kit (Cytoskeleton) (Tang and Gunst, 2004). Briefly, 30 min after wounding, astrocyte monolayers (5.5 cm^2^) were washed once in ice-cold PBS and rapidly mixed with 1 ml of ice-cold lysis buffer [50 mM Tris, 10 mM MgCl_2_, 0.5 M NaCl and 2% Igepal, protease inhibitor cocktail (pH 7.5)] to yield a protein concentration of 400-600 µg/ml. An aliquot was further taken to perform both BCA protein assay and Cdc42 immunoblot on the total Cdc42 fraction. The mix was then clarified by ultracentrifugation (100,000 ***g*** at 4°C for 1 min), and the supernatant was incubated with GST-p21-activated kinase (PAK) binding domain (PBD) affinity beads at 4°C for 1 h on a rotator. The beads were next ultracentrifugated (30,000-50,000 ***g*** at 4°C for 1 min), washed and resuspended in wash buffer [25 mM Tris, 30 mM MgCl_2_, 40 mM NaCl, protease inhibitor cocktail (pH 7.5)]. After a second step of ultracentrifugation (30,000-50,000 ***g*** at 4°C for 3 min), 10-20 µl of Laemmli 2× was added to resuspend the beads, and the proteins were boiled for 2 min. A western blot for Cdc42 was finally performed on the total and PBD-bound sample fractions with an appropriate dilution of Cdc42 antibodies (1:150). The intensity of activated GTP-bound Cdc42 was normalized to the total Cdc42 levels, and GAPDH (total) as well as Ponceau staining (GTP) were used as loading controls.

### Isolation of synaptosomal crude membrane fraction

For the isolation of synaptosomal crude membrane fractions, freshly dissected hippocampi were first lysed in 500 µl of ice-cold homogenization buffer (0.32 M sucrose, 10 mM HEPES, 2 mM EDTA, containing a freshly prepared cocktail of protease inhibitors) using a mechanical Potter-Elvehjem homogenizer. The homogenates were then centrifuged at 1000 ***g*** for 15 min at 4°C and the pelleted nuclear fractions discarded. The supernatants were further centrifuged at 200,000 ***g*** for 30 min at 4°C and the new pellets were washed and resuspended in ice-cold homogenization buffer, while the supernatant crude cytosols were withdrawn. Finally, crude membrane pellets were centrifuged one last time at 200,000 ***g*** for 30 min at 4°C and resuspended in 100 µl of HEPES lysis buffer [50 mM HEPES, 2 mM EDTA (pH 7.4), containing a freshly prepared cocktail of protease inhibitors]. Samples were briefly sonicated and the protein concentration of synaptosomal crude membrane fraction was next determined using a Bradford protein assay, and further processed for immunoblot as described above.

### AAV production and injection

For AAV *in vivo* gene transfer, a transgene composed of GFP and Cx30 cDNA separated by a P2A sequence in a single open reading frame was placed under the control of a GFAP-specific promoter in an AAV shuttle plasmid containing the inverted terminal repeats (ITR) of AAV2. The control vector expressed GFP only. Pseudotyped serotype 9 AAV particles were produced by transient co-transfection of HEK-293T cells, as previously described (Berger et al., 2015). Viral titers were determined by quantitative PCR amplification of the ITR on DNase-resistant particles and expressed as vector genomes vector genome per ml (vg/ml).

*Cx30*^−/−^ mice (2-week-old) were anesthetized with a mixture of ketamine (95 mg/kg; Merial) and xylazine (10 mg/kg; Bayer) in 0.9% NaCl and placed on a stereotaxic frame under body temperature monitoring. AAVs were diluted in PBS with 1% BSA at a concentration of AAV-GFP 1.2×10^13^ vg/ml and AAV-GFP-Cx30 0.5 x10^13^ vg/ml, and 1 µl of virus was stereotaxically injected bilaterally into the hippocampi at a rate of 0.2 μl/min, using a 29-gauge blunt-tip needle linked to a 2 µl Hamilton syringe (Phymep). The stereotaxic coordinates to Bregma were: antero-posterior, −1.7 mm; lateral: ±1.5 mm; and dorso-ventral, −1.7 mm. At the end of the injection, the needle was left in place for 5 min before being slowly removed. The skin was sutured, and mice recovery was checked for the next 24 h. After 2 weeks, the mice were sacrificed and processed for immunohistochemistry.

With the dose of AAVs and injection conditions used in this study, no astrogliosis was observed. Astroglial morphology was analyzed at a distance of ∼150–200 μm from the injection site and the images were taken at the same distance.

### Gap-junction coupling in hippocampal slices

Acute transverse hippocampal slices (400 µm) were prepared as previously described (Pannasch et al., 2014). Slices were transferred to storage chamber containing artificial cerebrospinal fluid (aCSF) (in mM: 119 NaCl, 2.5 KCl, 2.5 CaCl_2_, 1.3 MgSO_4_, 1 NaH_2_PO_4_, 26.2 NaHCO_3_ and 11 glucose, saturated with 95% O_2_ and 5% CO_2_) for at least 1 h at room temperature before the experiments. Slices were transferred in a submerged recording chamber mounted on an Olympus BX51WI microscope equipped for infrared-differential interference (IR-DIC) microscopy and were perfused with aCSF (2 ml/min). Whole-cell patch-clamp recordings were obtained from *s.r.* astrocytes using 4-6 MΩ glass pipettes filled with (in mM) 105 K-gluconate, 30 KCl, 10 HEPES, 10 phosphocreatine, 4 ATP-Mg, 0.3 GTP-Tris and 0.3 EGTA (pH 7.4, 280 mOsm). Astrocytes were patched at similar distances from the *stratum pyramidale* (∼200 µm) and were identified by their small somata, low input resistance and resting membrane potential, passive membrane properties (linear IV relationship), lack of action potential and extensive gap junctional coupling. For intercellular dye coupling experiments, the internal solution contained biocytin (7 mg/ml), which diffused passively in astrocytes patched in current-clamp mode for 30 min. Recordings were acquired using a MultiClamp 700B amplifier (Molecular Devices), digitized at 10 kHz, filtered at 2 kHz, stored and analyzed on a computer using Clampex10.3 and Clampfit10.3 Software (Molecular Devices). Biocytin was obtained from Sigma, and all other chemicals were from Tocris.

### Statistics

All data are expressed as mean±s.e.m. Data were obtained from at least three independent experiments that were performed in astrocyte cultures, hippocampal slices or mice, according to the type of experiment performed (*in vitro*, *ex vivo* and *in vivo*, respectively). For cellular analysis performed in astrocyte cultures or *in vivo*, *n* refers to the number of cells analyzed from five fields per independent experiment. For biochemical experiments performed in astrocyte cultures, *n* refers to the number of cultures; when performed *in vivo*, *n* refers to the number of mice. For intercellular coupling experiments in astrocyte cultures, *n* refers to the number of cultures; when performed *ex vivo*, *n* refers to the number of hippocampal slices. Statistical significance for within-group comparisons was determined by one-way ANOVA (followed by Dunnett's post-test), whereas unpaired *t*-tests were used for between-group comparisons, unless otherwise stated. Statistical analysis was performed in GraphPad InStat.

## Supplementary Material

Supplementary information

Supplementary information
