# Supplementary material for: Connexin 30 controls astroglial polarization during postnatal brain development
Source: Development. 2018 Feb 15;145(4):dev155275. doi: 10.1242/dev.155275 (PMC5869003; doi:10.1242/dev.155275)
Supplement: Supplementary information [file develop-145-155275-s1.pdf]

Supplementary Material

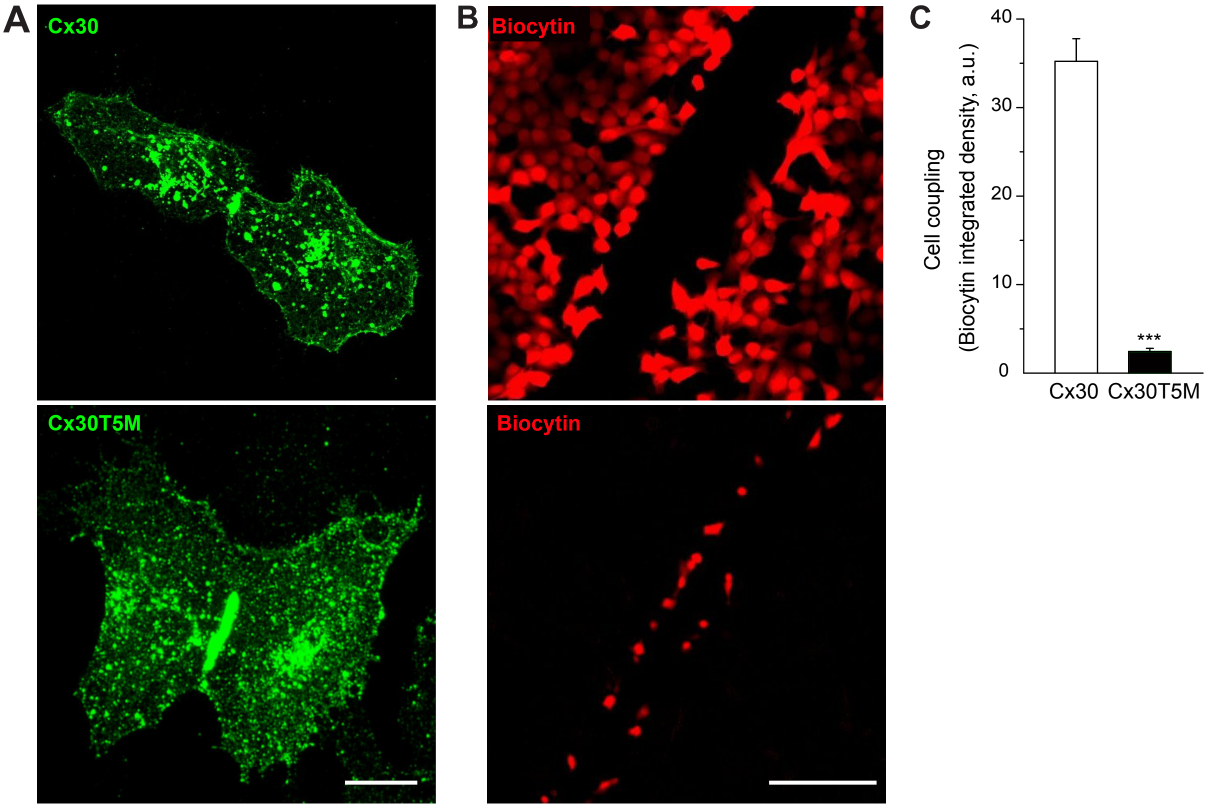

Figure S1. The Cx30T5M mutation leads to a defective intercellular biochemical coupling but intact membrane targeting. A. Immunocytochemical labeling for Cx30 (green) in transfected cultured cells shows proper Cx30T5M membrane targeting and GJ plaque formation, as compared to Cx30. Scale bar: 10 µm. **B, C.** The Cx30T5M mutation causes an inhibition in gap junctional biochemical coupling compared to Cx30 transfected cells (Cx30: 35.22 ± 2.55, n = 4 cultures, Cx30T5M: 2.44 ± 0.36, n = 4 cultures), as revealed by biocytin diffusion (red) following scrape-loading. Scale bar: 100 µm. Asterisks indicate statistical significance (***$p < 0.001$).

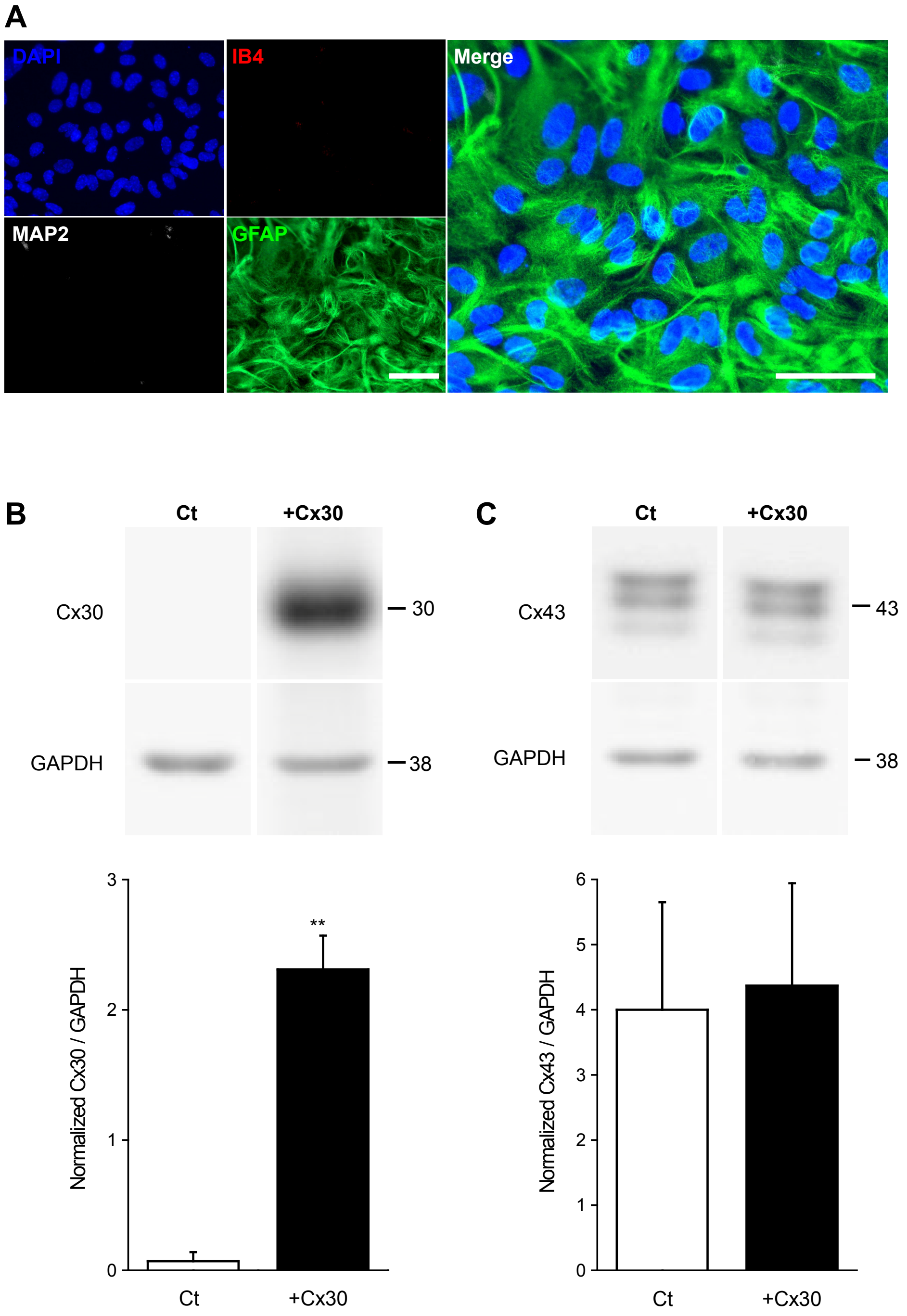

Figure S2. Cx expression in cultured astrocytes. **A.** Purity of astroglial cultures was assessed by immunocytochemistry for GFAP, MAP2, IB4 and DAPI. Sample images showing that the cultured cells are positive for the GFAP marker. Scale bars: 50 µm. **B.** Immunoblots showing Cx30 and Cx43 protein levels in astrocytes transfected (+Cx30) ot not (Ct) with the Cx30 plasmid. GAPDH was used as loading control. **C.** Quantitative analysis of the linear intensity profile of Cx30 and Cx43 (n = 5 cultures) normalized to that of GAPDH. Asterisks indicate statistical significance (**$p < 0.05$).

Development • Supplementary information

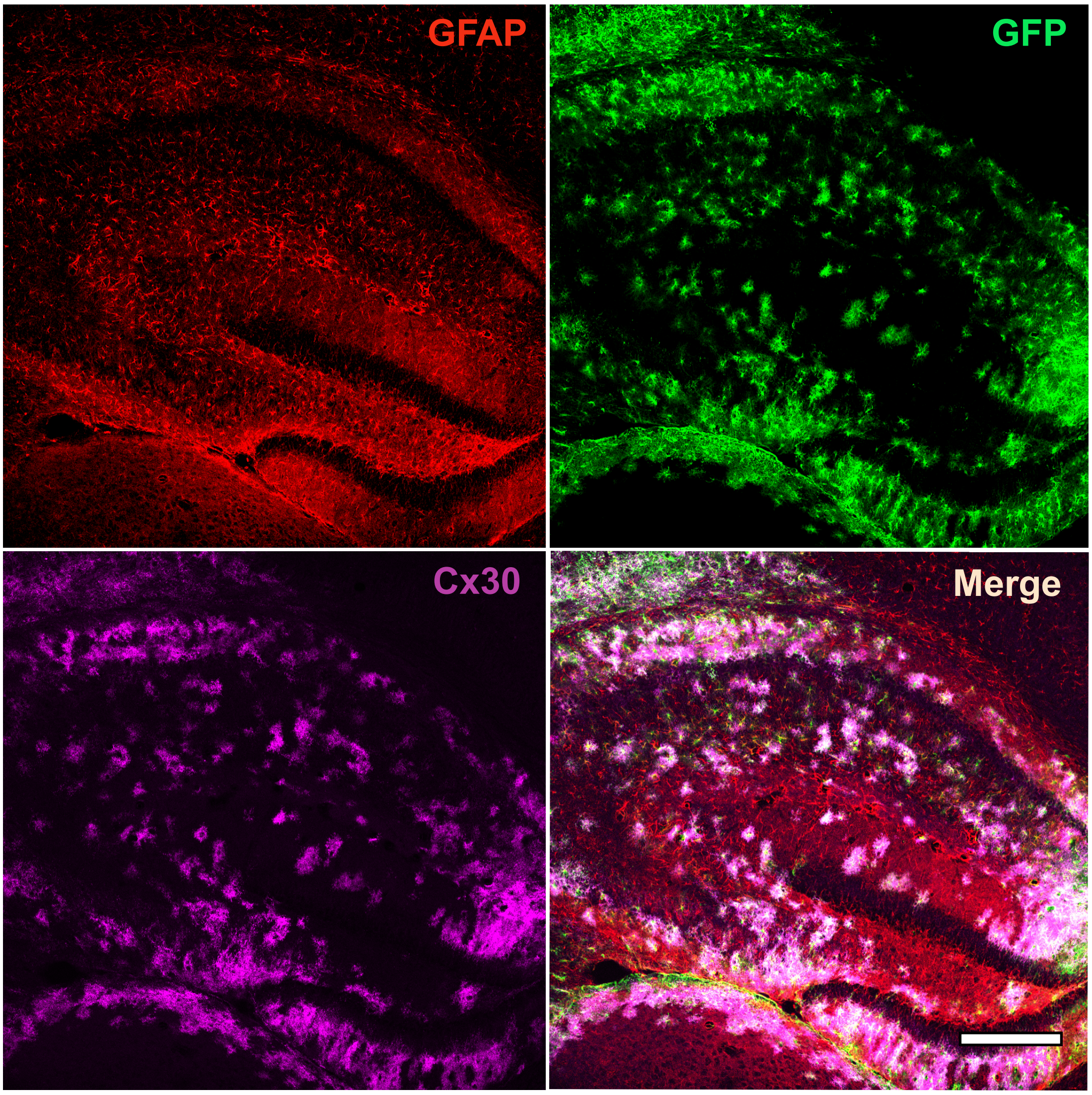

Figure S3. ***In vivo*** **rescue of astroglial Cx30 expression in the hippocampus of** ***Cx30***$^{-/-}$ **mice.** Cx30 protein was expressed *in vivo* specifically in astrocytes from *Cx30*$^{-/-}$ mice by injection in the hippocampus at P15 of an AAV encoding Cx30 with a GFP reporter. GFAP, Cx30 and GFP labeling in hippocampal sections from *Cx30*$^{-/-}$ mice (1-month-old) infected with AAVs at P15, showing local induction of Cx30 and GFP in astrocytes throughout the hippocampus. Scale bar: 250 µm.

Development • Supplementary information
